# Intravascular embolization with spinal decompression and vertebral shaping for acute cauda equina syndrome from aggressive vertebral hemangioma: a case report and literature review

**DOI:** 10.3389/fsurg.2025.1647623

**Published:** 2025-09-11

**Authors:** Qing-Quan Chen, Hong-Shen Wang, Hui Wang, Jin-Shui Chen, Jie Xiao, Xiu Yang

**Affiliations:** Spinal Ward, The 900th Hospital of PLA Joint Logistic Support Force, Fuzong Clinical Medical College of Fujian Medical University, Fuzhou, China

**Keywords:** aggressive vertebral hemangioma, embolization therapeutic, spinal cord compression, vertebroplasty, multidisciplinary approach

## Abstract

**Objective:**

To evaluate the clinical outcomes of a multidisciplinary approach for the treatment of aggressive vertebral hemangioma with acute cauda equina compression.

**Case description:**

A 37-year-old female patient with aggressive vertebral hemangioma presented with sudden loss of muscle strength in both lower limbs (grade I–II) and difficulty in urination and defecation. Magnetic resonance imaging and digital subtraction angiography confirmed a vascular tumor within and around the L4 vertebra, causing cauda equina compression. The treatment involved staged vascular embolization (of the third lumbar artery and branches of the sacral artery) combined with L4 vertebroplasty, laminectomy decompression, and pedicle screw fixation. Postoperative pain was immediately relieved. After 3 months of rehabilitation, muscle strength in both lower limbs recovered to grade 3, with significant improvement in spontaneous urination and defecation. Imaging studies showed complete relief of spinal canal compression.

**Conclusions:**

Aggressive spinal hemangioma may require multidisciplinary collaboration, and staged vascular embolization combined with spinal decompression and stabilization surgery can effectively improve neurological function. Early intervention is crucial for achieving favorable outcomes.

## 1 Introduction

Vertebral hemangioma (VH), first described by Perman in 1926 (1), is a common benign vascular lesion, accounting for 2%–3% of all spinal tumors (2). Based on clinical presentation, VHs can be categorized as latent, aggressive, or symptomatic, with the vast majority being asymptomatic, quiescent lesions (3). This report describes a 37-year-old woman presenting with low back pain due to an aggressive lumbar VH, accompanied by progressive cauda equina and lower limb neurological deficits.

The optimal treatment for aggressive VH remains unclear, and authoritative guidelines have not yet been established. Currently, various treatments are used to achieve desired therapeutic effects, including corticosteroid therapy, endovascular embolization, radiotherapy, and surgical resection. The primary goal is to restore spinal cord function and minimize damage to the spinal cord nerve function caused by the lesion. This case involved an adult with a sudden onset of lumbar aggressive VH. The condition was successfully treated using a combination of imaging, interventional, and surgical approaches, offering new insights into its clinical management.

## 2 Case description

### 2.1 Initial presentation

**Figure 1:**
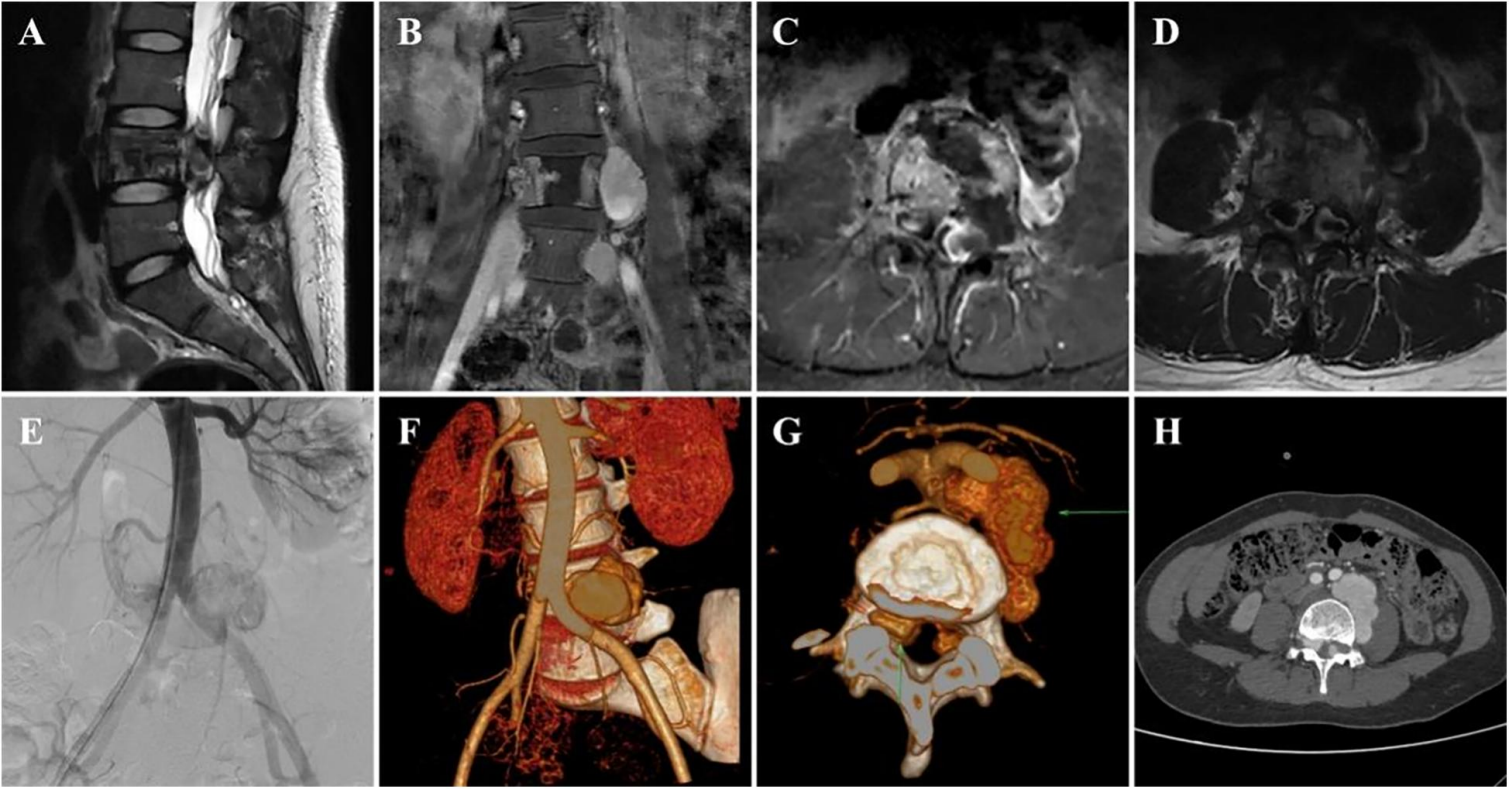
Preoperative imaging data of the patient. **(A–D)** Preoperative MRI images **(E)**, preoperative DSA image, and **(F–H)** preoperative CTA image.

A 37-year-old woman sought treatment from a spinal surgeon because of sudden pain and weakness in both legs and a 6-day history of inability to urinate or defecate. Physical examination revealed decreased strength in the key muscles of both legs: grade II for the iliopsoas, grade II for the quadriceps femoris, and grade I for the tibialis anterior and gastrocnemius. The patient also exhibited reduced sensation in the saddle area. Magnetic resonance imaging (MRI) revealed multiple masses of varying sizes in the L4 vertebra, spinal canal, and surrounding tissues, which appeared as uniformly high signals on T2-weighted images and short TI inversion recovery (STIR) sequences, indicating hemangiomas (Figures 1A–D). The extramedullary subdural hemangioma exhibited “double-eyed” signs and signs of vascular leakage (Figure 1D). Computed tomography angiography (CTA) and digital subtraction angiography (DSA) confirmed an L4 segment arteriovenous fistula with blood supply from the third lumbar artery and a branch of the sacral middle artery (Figures 1E,F), along with intraspinal hemangioma masses (Figure 1H). The patient was diagnosed with aggressive VH and incomplete paraplegia.

### 2.2 Treatment

During the first phase of the vascular intervention embolization, two femoral artery puncture super-selective embolization procedures targeting the lumbar artery and sacral artery branches at the third lumbar vertebra level were performed. However, the patient's symptoms persisted, and neurological function did not improve within 24 h post-procedure. The lack of symptomatic improvement following super-selective embolization suggested that spinal cord compression persisted.

During the second-stage surgery, the patient underwent spinal canal decompression and pedicle fixation combined with vertebroplasty. During the procedure, the patient was placed in a prone position under general anesthesia. The L4 vertebral body was located, a disinfected bandage was spread, and a posterior median lumbar approach incision was made to expose the L3–5 area. Under the guidance of a C-arm machine, the pedicles on both sides of L3 and L5 were successively located, opened, and tapped. The pedicle screws were screwed in for fixation, and a pre-bent titanium rod was installed. Vertebral fixation surgery was performed first to minimize the impact on vertebral stability as much as possible. Subsequently, an L4 total laminectomy and decompression were performed, the ligamentum flavum was removed, and the dural sac was pulled to one side using a nerve hook to explore the spinal canal. Malformed and tortuous blood vessels were observed in the spinal canal. Sufficient hemostasis was achieved after resection of the arteriovenous malformation. A polymethyl methacrylate (PMMA) injection pusher was inserted along each side of the L4 pedicle. Subsequently, 2 ml of bone cement was injected into each side of the vertebral body under direct vision, combined with C-arm machine fluoroscopy. After the bone cement solidified, fluoroscopy was performed again to confirm the position of the pedicle screw and PMMA. The area was subsequently rinsed with saline, one drainage tube was placed, and the wound was sutured layer by layer.

### 2.3 Postoperative care and rehabilitation treatment

**Figure 2:**
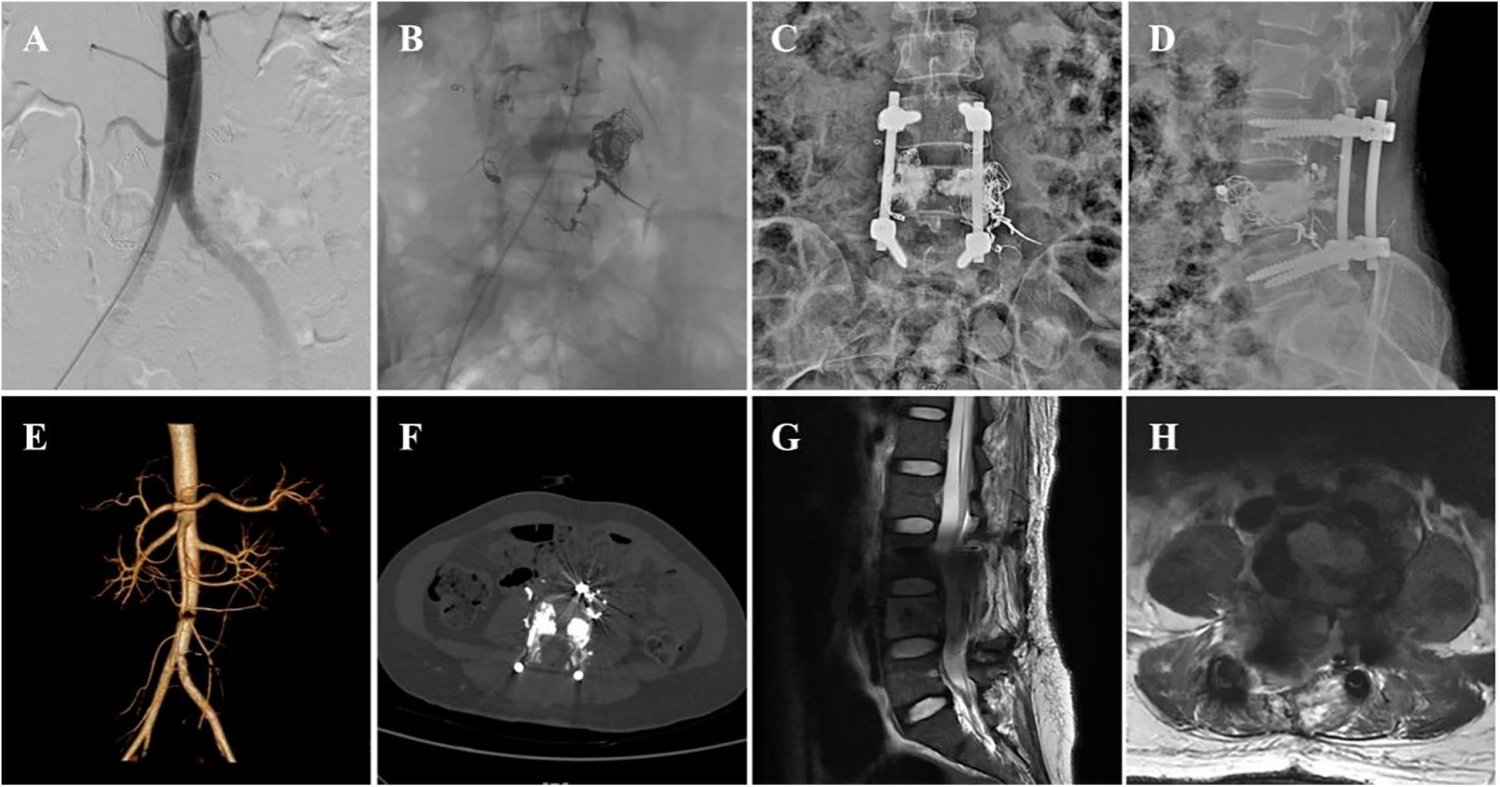
Postoperative imaging data of the patient. **(A,B)** postoperative DSA images, **(C,D)** postoperative x-ray anteroposterior and lateral views, **(E,F)** postoperative CTA images, and **(G,H)** postoperative MRI images.

The patient's pain in both lower extremities was completely relieved postoperatively. Drug treatment included methylprednisolone and mecobalamin for nerve repair and ibuprofen and pregabalin for pain management. Imaging reexamination with DSA (Figures 2A,B), x-ray (Figures 2C,D), CTA (Figures 2E,F), and MRI (Figures 2G,H) scans confirmed that the intraspinal and paraspinal aneurysms had completely disappeared. The rehabilitation treatment plan (implemented 2 weeks after surgery) included lower extremity motor function training, balance ability exercise, medium frequency pulsed electrical stimulation therapy, pneumatic therapy, and biofeedback training for urinary and fecal functions.

### 2.4 Postoperative follow-up

The pain in both lower limbs disappeared postoperatively, and rehabilitation training was initiated 2 weeks later. After 3 months of rehabilitation, follow-up examinations revealed that the lower-limb muscle strength recovered to grade Ⅲ and that the residual bladder urine volume was <50 ml. Moreover, MRI revealed complete relief of spinal canal compression with no recurrence.

## 3 Discussion

VHs are the most common benign spinal tumors in adults, accounting for 20%–30% of all benign spinal neoplasms (4). The majority are asymptomatic, with only approximately 5% becoming symptomatic (5). In most symptomatic patients, symptoms are limited to low back pain; the development of compressive neurological symptoms is considerably less common (6). VHs most frequently originate in the lower thoracic or lumbar spine, with 20%–30% of patients exhibiting multiple lesions (7). When VHs progress to an “aggressive” form, characterized by extraosseous extension, they can cause spinal cord compression, resulting in neurological symptoms and pain. Four mechanisms of spinal cord and nerve root compression have been proposed: (1) hypertrophy or bulging of the posterior vertebral cortex caused by the hemangioma, (2) epidural extension of the hemangioma through the cortex, (3) compression fracture of the involved vertebral body, and (4) epidural hematoma (8).

MRI can reveal changes in intramedullary signals and most blood vessels, making it safer than invasive angiography using contrast agents (9). CT demonstrates the typical feature of thickened vertebral trabeculae, appearing as hyperdense areas, also known as the “polka-dot sign” or “salt-and-pepper sign” (10). Therefore, MRI and CT scans are crucial for diagnosing aggressive VH. Based on the presence of symptoms and imaging findings, Boriani et al. classified VHs into four groups (11): Type I: Latent, with minimal bone destruction and asymptomatic. Type II: Active, with bone destruction and pain. Type III: Aggressive, asymptomatic lesion with epidural and/or soft tissue extension. Type IV: Aggressive lesion with epidural and/or soft tissue extension, causing neurological deficits.

Aggressive and symptomatic VHs frequently occur in the thoracic spine due to the limited space available for the spinal cord in this region (12). Although the lesion in this case was located in the lumbar segment, aggressive VH should also be strongly suspected in patients presenting with acute symptoms of spinal cord or cauda equina compression, particularly when accompanied by characteristic imaging findings (MRI/CT). These include the “honeycomb” or “corduroy” pattern typical of VH, pedicle erosion, cortical expansion, vertebral body collapse, irregular vertical trabeculations associated with lytic areas of varying sizes, and intra- and extravertebral vascular anomalies. This case presented as an aggressive VH of the lower lumbar spine, primarily affecting the cauda equina rather than spinal cord functions. Given the communication between the vascular tumor within the spinal canal and vertebral body, the lower-limb symptoms may have resulted from venous hypertension and vascular stealing along the spinal cord.

Since the overwhelming majority of VHs are asymptomatic and discovered incidentally, they do not require treatment. However, aggressive VHs can lead to spinal cord compression, vertebral destruction, or neurological deficits. Surgical intervention is typically reserved for patients presenting with severe neurological deficits or significant instability (13). Previous literature has reported that the primary treatment modalities for treating aggressive VHs are interventional embolization and surgical resection. Treatment aims to restore spinal cord function, maintain spinal stability, and reduce pathological changes that damage spinal cord nerve function. Embolization is a minimally invasive treatment often preferred in many cases (14). In a series of 26 patients, Premat et al. (15) demonstrated that embolization combined with vertebroplasty is effective and safe for treating pain associated with aggressive VH but is less effective in resolving motor deficits. However, achieving curative treatment is challenging. Therefore, endovascular surgery aims to prevent bleeding and neurological deterioration. After multidisciplinary discussions, we collaborated with the radiology department for two interventional treatments, but the results were unsatisfactory. This is similar to the results reported in the literature. The poor embolization effect may be due to ligation of spinal arteriovenous malformations providing only temporary benefits and leading to multiple collateral channels, resulting in treatment failure (16). Complete endovascular occlusion is challenging and more suitable for specific lesions without intramedullary involvement (17).

Determining whether managing aggressive VH requires further surgery after addressing intravascular embolism is challenging. Surgical intervention, as one of the treatment modalities for aggressive VHs, achieves mass removal with instrumentation and fusion, decompression of neural structures, and spinal stabilization (18). Primary surgical objectives should include interrupting the arteriovenous shunt, preserving the main drainage vein, and decompressing the spinal cord (Prasad et al., 2022 (19). Surgery may be essential for treating aggressive VHs with mechanical compression. Kato et al. (20) performed a combination of preoperative transarterial embolization and total resection for aggressive VHs with extraosseous extension causing spinal cord compression in all five reported cases. Long-term follow-up resulted in satisfactory outcomes. Gabay et al. (21) reported a case of quadriplegia due to cervical arteriovenous malformation and spinal canal stenosis, where symptoms improved after vascular embolization with spinal stabilization surgery. Gala et al. (22) believed that surgical decompression is necessary when vertebral vascular tumors cause progressive neurological deficits. Preoperative arterial embolization is needed if malformed vessels are highly distributed. Symptoms did not improve after embolization, indicating the need for combined surgery to relieve compression. The operation aimed to interrupt the arteriovenous shunt, retain the drainage vein and spinal cord decompression, and maintain vertebral stability. Studies indicate that given the characteristics of aggressive VHs, minimally invasive surgical approaches are necessary for tumor management, and preoperative embolization significantly reduces intraoperative blood loss (23).

The fundamental principle of surgical management for aggressive VHs is adequate decompression of the dural sac, followed by stabilization with fusion of the involved vertebral segment (13). Moreover, percutaneous injection of bone cement (PMMA) serves as an effective approach to alleviate pain associated with spinal hemangiomas (24). Laminectomy, pedicle screw fixation, and PMMA vertebral body augmentation were performed in our case. Preoperative embolization helps reduce vascular distribution for better surgical treatment and also reduces PMMA venous leakage risk. During surgery, the responsible lamina was removed for complete decompression of the spinal canal, while preserving the bilateral facet joints to minimize impact on spinal stability. Pedicle screws fixed the L3 and L5 vertebrae, and PMMA was slowly injected into the L4 vertebra (outer 1/3) under the gaze of the cameras using a bilateral approach to fill the vertebral body, ensuring uniform distribution and preventing leakage. In such cases, arterial embolization and vertebral decompression fixation surgery may be insufficient to alleviate symptoms. Therefore, we recommend addressing vascular tumors of the vertebral body to prevent collateral circulation. For vertebral vascular tumor treatment, procedures such as vertebral augmentation, sclerotherapy, and embolization can be used alone or with surgery (9). Wang et al. (3) found that in a study of 39 patients with invasive vertebral vascular tumors, combining surgical decompression with intraoperative PMMA-filling vertebral augmentation helped reduce bleeding as well as the likelihood of postoperative recurrence. Mousavi et al. (25) treated a patient with an invasive vertebral vascular tumor, bilateral lower-limb paralysis, and sphincter dysfunction by performing selective arterial embolization plus laminectomy for decompression and PMMA vertebral augmentation reconstruction. The patient's pain immediately improved, and neurological symptoms gradually improved. Allegretti et al. (26) conducted a retrospective study of cases in which intraoperative vertebral body augmentation could be used to stabilize the vertebra and relieve pain in patients with compressive myelopathy, including VHs, who are candidates for decompressive laminectomies. Therefore, the use of PMMA to fill the vertebral body was considered feasible. Clinically, the puncture needle for a unilateral pedicle approach must reach 1/4–1/3 of the anterior edge of the vertebral body, with the needle tip reaching or approaching the midline of the vertebral body to ensure uniform PMMA-filling. One common complication of vertebral body augmentation surgery is PMMA leakage into adjacent structures, occurring in the spinal canal or veins (27). Since patients have paravertebral arteriovenous malformations, PMMA leakage can lead to catastrophic complications. Studies have shown that injecting PMMA into the central vertebral body region can cause leakage into the basilar venous plexus, which is a surgical risk factor since the central region is the main basilar venous plexus pathway (28). Baek et al. (29) suggested that, in order to prevent intradural PMMA leakage, the needle tip should not penetrate the medial pedicle wall. Therefore, we decided to inject PMMA intraoperatively within 1/3 of the lateral vertebral body region. We believe bilateral puncture poses less risk for PMMA venous leakage, as each side receives less PMMA and has a smaller puncture angle, reducing venous system interference. The patient's lower-limb symptoms were significantly relieved postoperatively. As Jiang et al. (30) and Wang (3) stated, surgery is required to eliminate space-occupying effects in refractory cases, demonstrating this protocol's necessity.

Based on these findings, a multidisciplinary collaboration (spinal surgery and interventional radiology) is essential for treating aggressive VHs. Combined interventions prevent symptom progression and reduce neurological sequelae by alleviating mass effect, venous hypertension, and vascular steal along the spinal cord (31). The treatment principle of “blood flow control-nerve recovery-structural stability” was followed with staged embolization: achieving complete intravascular embolization of the fistula without affecting spinal cord perfusion, preventing neurological deterioration and reducing intraoperative bleeding risk; vertebral body shaping and spinal canal decompression: removing the embolized fistula and abnormal vascular system, eliminating mass effect, relieving compression, restoring spinal cord function, and maintaining stability; PMMA-filled vertebral body shaping: hardening the vascular plexus within the vertebra to prevent collateral circulation formation and enhance anterior column support, providing vertebral stability. The combination of interventional radiology and spinal surgery balances vascular control and nerve decompression, thereby shortening the treatment period.

In our case, the better prognosis was linked to our surgical approach as well as other factors, including the extramedullary location of the spinal vascular malformation, the lesion location at the lower spinal canal level, and the strong compensatory ability of the cauda equina. The literature reports that not all neurological injuries caused by vascular malformation improve with long-term follow-up after treatment. Muscle strength recovery has been reported to be the fastest, while sphincter function recovery has been reported to be the slowest (32). Moreover, skin sensory abnormalities often persist during follow-up. Given that malformations are often extensive and span multiple spinal levels, achieving curative treatment is challenging, and recurrence is common. Therefore, management is typically palliative, aimed at preventing further neurological deterioration. The 3-month follow-up period in this study is insufficient to fully assess the long-term prognosis of aggressive VHs. A longer observation period is required to evaluate the risk of vascellum recurrence (e.g., collateral circulation formation), long-term complications of internal fixation or bone cement, and the stability of neurological recovery—particularly sphincter function. Thus, early diagnosis, timely intervention, and annual postoperative follow-up with MRI or CT are strongly recommended.

## 4 Conclusion

Aggressive VH is a congenital vascular anomaly impacting the spine, spinal cord, and nerves within the same vertebral segment. In this case, despite an acute onset, the patient recovered well, indicating that early MRI with CT examination should be performed for suspected cases to avoid delayed diagnosis. For aggressive VH with acute cauda equina compression, staged endovascular embolization, laminectomy decompression, and vertebral body formation and fixation can effectively improve neurological function. Limiting PMMA to the external third of the vertebral body is critical. Multidisciplinary collaboration involving vascular surgery, neurosurgery, spinal surgery, and interventional radiology is essential for developing a personalized treatment plan, weighing neurological function against intervention risks to minimize permanent damage.

## Data Availability

The original contributions presented in the study are included in the article/Supplementary Material, further inquiries can be directed to the corresponding author/s.
